# Is the cat an important reservoir host for visceral leishmaniasis? A systematic review with meta-analysis

**DOI:** 10.1590/1678-9199-JVATITD-2019-0012

**Published:** 2019-06-10

**Authors:** Shabnam Asfaram, Mahdi Fakhar, Saeed Hosseini Teshnizi

**Affiliations:** 1 Student Research Committee, Department of Parasitology, School of Medicine, Mazandaran University of Medical Sciences, Sari, Iran.; 2 Toxoplasmosis Research Center, Department of Parasitology, School of Medicine, Mazandaran University of Medical Sciences, Sari, Iran.; 3 Infectious and Tropical Diseases Research Center, Hormozgan University of Medical Sciences, Bandar Abbas, Iran.

**Keywords:** Feline leishmanial infection, Global prevalence, Diagnostic tests, Systematic review, Meta-analysis

## Abstract

In recent years feline leishmanial infections (FLI) have been studied more than ever before in various parts of the world. However, evidence-based knowledge on FLI has remained unavailable. The main objectives of this study were to investigate the status of felines infected by *Leishmania* spp. worldwide. Data were extracted from 10 available databases over the period of 1982 to 2017. Overall, 78 articles fulfilled the inclusion criteria and were used for data extraction in this systematic review. The overall FLI prevalence by both serological and molecular methods was estimated at 10% (95% CI: 8%-14%). In Italy, both the seroprevalence (24 %) and PCR prevalence (21 %) were found to be higher than in other countries. The most common diagnostic test used was the indirect fluorescent antibody test (38.5%). Studies on mixed-breed felines were more common than those on other breeds, while the most common parasite species was *L. infantum* (63%). Our findings suggest that cats act as primary and/or secondary reservoir hosts in the transmission of the *Leishmania* spp. to humans and also to dogs, by sandflies, at least in endemic foci. Moreover, available data confirm the enzootic stability situation of FLI in several countries including some in Europe.

## Background

The leishmaniases are neglected protozoal diseases caused by *Leishmania* spp. that occur in 98 countries [1], affecting 1.2 million in the form of cutaneous leishmaniasis (CL), and 400,000 in the form of visceral leishmaniasis (VL), leading to approximately 40,000 deaths per year [2]. The main route of VL transmission is through the bite of vectors infected with *Leishmania donovani* (*L. donovani)* complex, mainly *Leishmania infantum/chagasi* (*L. infantum/chagasi*). Both domestic and wild animals may serve as host reservoirs of *Leishmania* spp. [3]. Dogs are the main reservoir hosts of *L. infantum/chagasi* but sandflies, as the natural vectors of *Leishmania* spp., may also feed on the blood of cats [4]. Therefore, cats infected with the *L. donovani* complex may be urban reservoirs of VL and transmit the protozoan to other sandflies [5, 6]; therefore, cats are potential reservoirs of this zoonotic VL disease. Studies on feline leishmanial infection (FLI) are limited and several aspects of the disease in cats are still unclear [7]. Recently, reports of FLI have increased dramatically, achieving a prevalence of up to 60% in certain cat populations [8]. The most common clinical signs reported in FLI include lymphadenomegaly, splenomegaly, weight loss, anorexia, as well as cutaneous, mucocutaneous and ocular lesions [8]. However, in endemic regions such as Mediterranean countries, the subclinical feline infection *L. infantum/chagasi* is common, whereas clinical illness is relatively uncommon [7-8].

 Identification of *Leishmania* amastigotes in aspirated samples of bone marrow, spleen and lymph node is specific and considered the gold standard method for diagnosing FLI. Feline vector-borne pathogens have been increasingly recognized worldwide based on serological and/or molecular epidemiological investigations [9,10]. Most epidemiological studies demonstrated the presence of anti-*Leishmania* antibodies in feline sera by means of different techniques such as indirect fluorescent antibody test (IFAT), enzyme-linked immunosorbent assay (ELISA) or western blot (WB) [10-17]. Polymerase chain reaction (PCR) is recommended preferentially over other diagnostic tests, especially when blood samples and other clinical samples contain a low parasitic burden [13, 16, 18,19]. However little is known in reference to their diagnostic performance in cats with FLI.

Although an effective treatment for symptomatic cats has not yet been established, oral allopurinol administration followed by subcutaneous glucantime has been frequently used as chemotherapy regimens in cats affected by FLI [7, 8, 20].

However, there is still no available evidence-based knowledge about various epidemiological aspects of FLI. Therefore, the purpose of this study was to determine the global status of the infection in cats and introduce currently used diagnostic laboratory methods.

## Methods

### Searching strategy

This systematic review was performed according to the guidelines of the Preferred Reporting Items for Systematic Reviews and Meta-Analysis (PRISMA) [21]. To determine the prevalence of FLI, 10 English and Iranian databases including Google Scholar, Pub Med, Science Direct, Web of Science, Scopus, Elm net, Magiran, Barakatkns (formerly Iran medex), Iran doc, and Scientific Information Database (SID) were searched from 1982 to 2017 (36 years). The relevant keywords including “*Leishmania* spp.”, “*Leishmania donovani*”, “*Leishmania infantum*”, “feline leishmaniasis”, “feline leishmaniosis”, “cat”, “molecular”, “PCR” , “serology”, “ELISA”, “IFAT” were chosen using medical subject headings terms (MESH). 

### Inclusion and exclusion criteria

**Figure 1.**
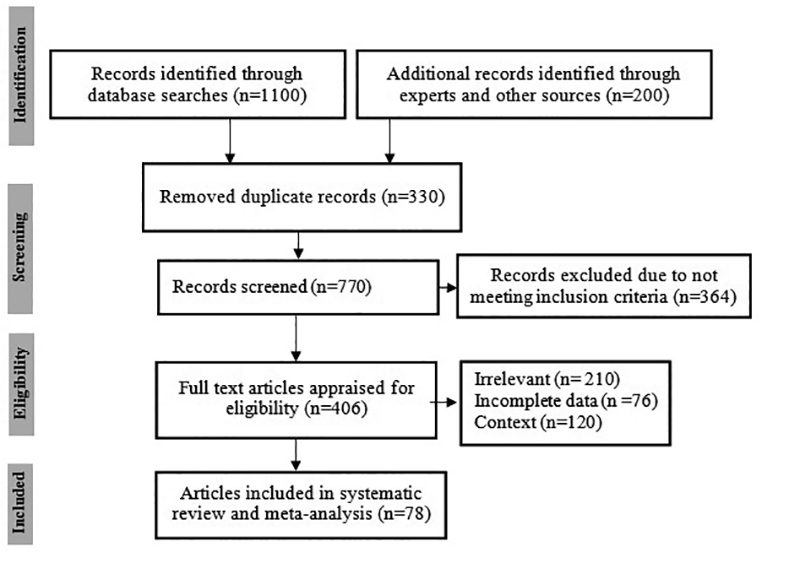
PRISMA flowchart showing the study design process.

Data were extracted from studies with at least one of the following inclusion criteria: cross-sectional and case-control studies corresponding to determining prevalence of leishmanial infections that evaluated the presence of FLI based on serological and molecular tests among all types of cats. Also, summaries of articles presented as proceedings at conferences, studies that contained no qualified data, experimental studies, review articles, duplicates, and case reports were excluded. The PRISMA flowchart of the study plan is shown in Figure 1. Out of the retrieved articles, 78 papers were eligible for inclusion in this systematic review and meta-analysis. The recorded data included author name, year of publication, country, type of cat, sample size, *Lieshmania* species, laboratory method, seroprevalence (%) and PCR prevalence and quality assessment. The above details were extracted separately by two researchers (SA and MF).

### Meta-analysis

For each study, the prevalence and standard error (SE= P(1−P) n ) were determined. We used forest plots to estimate pooled effect sizes and the effect of each study with 95% confidence intervals. The Cochran Q-test (p-value<0.1) and the I-squared index were employed to evaluate heterogeneity , with I 2 values between 25% and 50% as thresholds for low , between 50% and 75% for moderate, and above 75% for high heterogeneity. When heterogeneity was found, a random-effects model (Dersimonian-Laird model) was applied; if not, a fixed effects model (Mantel-Haenszel) was utilized to calculate overall effects. 

### Quality assessment

The quality of meta-analysis was evaluated with the STROBE checklist. A checklist including 22 items was considered for adequate reporting of observational studies. These items related to the article’s title, abstract, introduction, methods, results, and discussion sections. A score under 7 was defined as poor quality, 8 to 17 low, 18 to 28 moderate and more than more 28 high quality [22]. The mean score obtained via the STROBE checklist for 78 analyzed articles was 31, whereas 28 is considered high quality. Possible publication bias was explored using a funnel plot and Egger’s test, which evaluated whether the precision of studies was appropriate for the scale of their effect size. All data analyses were performed using the software Stata, v. 14 (Stata Corp LP, College Station, Texas, USA).

## Results

Seventy-eight (78) cross-sectional studies published from 1982 to 2017 (36 years) were included in this meta-analysis. Most studies (30.8%) were performed in Brazil (Table 1). The total number of cases was 12,635 (ranging from 8 to 1,101). In Italy, both the prevalence by seropositivity (24 %) and PCR positivity (21%) were found to be higher than in other countries. The overall seropraevalence of FLI in 4 European countries including Italy, Spain, Portugal, and Greece was estimated at 12.2% (see Table 2).

The most common diagnostic test was IFAT, used in 38.5% of studies. Among serological methods, WB and indirect hemagglutination test (IHT), being the least common diagnostic tests, were used in only 3.8 % of studies (see Table 1). 

The seroprevalence (15%) and PCR prevalence (23%) of FLI in mixed-type/ breed cats (defined as cats descending from two or more breeds) were higher than in other cat types/ breeds. Approximately 63% of Leishmania species were L. infantum, while the remainder frequently included *Leishmania* spp. (Table 2).

**Table 1:** Baseline characteristics of studies included in the meta-analysis of feline leishmanial infection

Author	Year of publication	Country	Type of cat	Sample size	*Leishmania species*	Lab test	Seropositive (%)	PCR positive (%)
Michael. SA [43]	1982	Egypt	stray	80	*Leishmania* spp.	IHAT	3.8	.
Morsy. TA [44]	1988	Egypt	stray	28	*Leishmania* spp.	IHAT	3.6	.
Bez. M [45]	1992	France	.	174	*Leishmania* spp.	IFAT	0.6	.
Morsy. TA [46]	1994	Egypt	mixed	60	*Leishmania* spp.	IHAT	10	.
Sherlock. IA [47]	1996	Brazil	.	53	*Leishmania* spp.	IFAT	0	.
Pennisi. MG [48]	1998	Italy	mixed	93	*Leishmania* spp.	IFAT	59.1	.
Ozon. C [29]	1998	France	stray	97	*L. infantum*	WB	12.4	.
Pennisi. MG [8]	2000	Italy	mixed	89	*Leishmania* spp.	IFAT, PCR	68.5	60.7
Simões-Mattos. L [49]	2001	Brazil	stray	84	*Leishmania* spp.	ELISA	10.7	.
Poli. A [31]	2002	Italy	domestic	110	*Leishmania* spp.	IFAT	0.9	.
Portús. M [50]	2002	Spain	domestic	117	*L. infantum*	ELISA	1.7	.
Zárate-Ramos. JJ [51]	2002	Spain	domestic	50	*L. infantum*	DAT	42	.
Vita. S [52]	2005	Italy	mixed	203	*Leishmania* spp.	IFAT, PCR	16.3	100
Solano-Gallego. L [37]	2007	Spain	mixed	445	*L. infantum*	ELISA	6.3	.
Martín-Sánchez. J [36]	2007	Spain	domestic	183	*L. infantum*	IFAT, PCR	70.5	25.7
Nasereddin. A [53]	2008	Israel	stray	104	*Leishmania* spp.	ELISA	6.7	.
Huebner. J [54]	2008	Greece	mixed	389	*Leishmania* spp.	IFAT	21.6	.
Tabar. MD [55]	2008	Spain	domestic	100	*L. infantum*	PCR	.	3
Ayllon. T [40]	2008	Spain	domestic	233	*L. infantum*	IFAT, PCR	4.29	0.4
Maia. C [56]	2008	Portugal	stray	23	*L. infantum*	IFAT, PCR	17.4	30.4
Da Silva. AV [42]	2008	Brazil	domestic	8	*L. infantum*	IFAT	25	.
Sarkari. B [57]	2009	Iran	stray	40	*L. infantum*	IFAT, DAT	27.5	.
Diakou. A [58]	2009	Greece	stray	284	*Leishmania* spp.	ELISA	3.9	.
Figueiredo. FB [11]	2009	Brazil	.	43	*Leishmania* spp.	IFAT, ELISA	2.4	.
Hatam. GR [59]	2010	Iran	domestic	40	*L. infantum*	PCR	.	7.5
Veronesi. F [60]	2010	Italy	mixed	95	*Leishmania* spp.	IFAT, PCR	9.5	5.3
Cardoso. L [35]	2010	Portugal	domestic	316	*L. infantum*	ELISA, DAT	2.8	.
Duarte. A [61]	2010	Portugal	stray	180	*L. infantum*	IFAT	0.6	.
Maia. C [62]	2010	Portugal	domestic	142	*L. infantum*	IFAT, PCR	1.3	20.4
Costa. TA [63]	2010	Brazil	.	200	*L. infantum*	ELISA	11.5	.
Dahroug. MA [64]	2010	Brazil	Puma concolor, Panthera onca, Leopardus pardalis	16	*L. infantum*	PCR	.	37.5
Bresciani. KD [65]	2010	Brazil	domestic	283	*Leishmania* spp.	IFAT	0	.
Sherry. K [36]	2011	Spain	mixed	105	*L. infantum*	ELISA, PCR	12.4	8.6
Millán. J [66]	2011	Spain	mixed	86	*L. infantum*	WB, PCR	15.7	25.6
Miró. G [67]	2011	Spain	.	20	*L. infantum*	IFAT	15	.
Vides. JP [12]	2011	Brazil	.	55	*L. infantum*	IFAT, ELISA	27.3	.
Da Silveira Neto. L [ 68]	2011	Brazil	.	113	*L. infantum*	ELISA	34.5	.
Coelho. WM [13]	2011	Brazil	.	70	*Leishmania* spp.	IFAT, ELISA	4.2	.
Coelho. WM [13]	2011	Brazil	.	52	*L. infantum*	PCR	.	5.8
Pennisi. MG [69]	2012	Italy	mixed	431	*Leishmania* spp.	IFAT, PCR	6.9	18.3
Ayllon. T [70]	2012	Spain	mixed	680	*L. infantum*	IFAT, PCR	3.7	0.6
Sobrinho. LS [14]	2012	Brazil	stray	302	*L. infantum*	IFAT, ELISA	15.23	.
Longoni. SS [17]	2012	Mexico	stray	95	*L. infantum*, *L. braziliensis*	ELISA, WB	31.6	.
Spada. E [71]	2013	Italy	stray	233	*Leishmania* spp.	IFAT, PCR	25.3	0
Vilhena. H [9]	2013	Portugal	domestic	320	*L. infantum*	PCR	.	0.3
Cardia. DF [72]	2013	Brazil	stray	386	*Leishmania* spp.	IFAT	0.5	.
Silva. RD [73]	2013	Brazil	.	153	*L. infantum*	ELISA	3.9	.
Chatzis. MK [10]	2014	Greece	domestic	100	*L. infantum*	IFAT, ELISA, PCR	11	41
Silaghi. C [74]	2014	Albania	stray	146	*Leishmania* spp.	IFAT, PCR	0.7	0
Miró. G [75]	2014	Spain	stray	346	*L. infantum*	IFAT, PCR	3.2	0
Maia. C [76]	2014	Portugal	mixed	649	*L. infantum*	PCR	.	9.9
Maia. C [76]	2014	Portugal	mixed	271	*L. infantum*	DAT	3.7	.
Nimsuphan. B [77]	2014	Bangkok	pet	237	*L. donovani*	DAT	0.8	.
Moreno.I [78]	2014	Spain	stray	43	*L. infantum*	IFAT	9.3	.
Dorbadam. SM [79]	2014	Iran	stray	50	*L. infantum*	DAT	2	.
Sousa. KC [80]	2014	Brazil	domestic	151	*L. infantum*	IFAT	6.6	.
Fatollahzadeh. M [81]	2014	Iran	.	65	*L. infantum*	DAT, PCR	23.0	0
Braga. AR [82]	2014	Brazil	domestic	50	*Leishmania* spp.	IFAT	4	.
Costa. AP [83]	2014	Brazil	domestic	52	*L. infantum*	IFAT	3.8	.
Braga. AR [84]	2014	Brazil	.	100	*Leishmania* spp.	IFAT, PCR	15	0
Nemati. T [85]	2015	Iran	.	65	*Leishmania* spp.	DAT	27.7	.
Maia. C [86]	2015	Portugal	mixed	271	*L. infantum*	DAT	3.7	.
Dincer. E [87]	2015	Turkey	.	22	*L. infantum*	PCR	.	4.5
Oliveira. TM [88]	2015	Brazil	.	52	*Leishmania* spp.	PCR	.	13.5
Spada. E [89]	2016	Italy	stray	90	*L. infantum*	IFAT, PCR	30	2.2
Figueiredo. FB [90]	2016	Brazil	domestic	34	*L. braziliensis*	ELISA	20.6	.
Persichetti. MF [91]	2016	Spain	.	42	*L. infantum*	IFAT, PCR	2.4	7.1
Can. H [16]	2016	Turkey	stray	1101	*L. infantum*, *L. tropica*	IFAT, ELISA, PCR	10.5	0.54
Persichetti. MF [15]	2017	Italy	.	161	*L. infantum*	ELISA, IFAT, WB	29.2	.
Otranto. D [92]	2017	Italy	.	330	*L. infantum*	IFAT, PCR	25.8	3.9
Mylonakis. ME [93]	2017	Greece	.	100	*L. infantum*	PCR	.	41
Mohebali. M [94]	2017	Iran	stray	103	*L. infantum*	DAT	24.3	.
Metzdorf. IP [95]	2017	Brazil	domestic	100	*L. infantum*	PCR	.	6
De Mendonça. IL [96]	2017	Brazil	domestic	83	*L. infantum*	ELISA	4	.
Lopes. AP [97]	2017	Portugal	domestic	102	*Leishmania* spp.	DAT	0	.
Poffo. D [98]	2017	Brazil	domestic	88	*Leishmania* spp.	PCR	.	0
Akhtardanesh. B [99]	2017	Iran	stray	60	*L. infantum*, *L. tropica*	ELISA, PCR	6.7	16.7
Benassi. JC [100]	2017	Brazil	mixed	108	*L. infantum*	PCR	.	1.85

**Table 2:** Subgroup meta-analysis for seroprevalence and PCR prevalence of Leishmania infection in cats

Characteristics	Factors	Seropositive				PCR positive			
		n	Prevalence (%) (95%CI)	I-square (%)	p	n	Prevalence (%) (95%CI)	I-square (%)	P<0.001
Country	Iran	6	17.0 (8.0-28.0)	83.8	0.06	3	6.0 (0.0-21.0)	-	P<0.001
	Egypt	3	6.0 (2.0-10.0)	98.3			NR	-	
	Greece	3	11.0 (2.0-26.0)	95.2			NE	-	
	Italy	10	24.0 (13.0-37.0)	97.1		7	21.0 (10.0-61.0)	99.5	
	Spain	12	12.0 (4.0-23.0)	97.7		8	6.0 (1.0-14.0)	96.7	
	Portugal	7	2.0 (1.0-4.0)	70.1		4	11.0 (2.0-26.0)	96.7	
	Brazil	17	8.0 (3.0-13.0)	93.7		7	5.0 (1.0-11.0)	85.3	
Diagnostic test	ELISA	12	9.0 (5.0-13.0)	87.8	P<0.001	-	-	-	-
	IHAT	3	6.0 (2.0-10.0)	97.5		-	-	-	
	IFAT	30	11.0 (6.0-17.0)	97.7		-	-	-	
	IFAT, ELISA	6	11.0 (7.0-16.0)	97.5		-	-	-	
	WB	3	14.0 (9.0-20.0)	96.6		-	-	-	
	DAT	9	10.0 (3.0-19.0)	94.9		-	-	-	
	PCR	12	7.0 (3.0-14.0)	93.5		-	-	-	
Breed of cat	Stray	22	10.0 (6.0-14.0)	95.2	0.46	7	2.0 (0.0-5.0)	90.5	P<0.001
	Domestic	17	7.0 (2.0-16.0)	97.6		9	8.0 (1.0-19.0)	96.8	
	Mixed	13	15.0 (8.0-24.0)	95.6		16	23.0 (4.0-50.0)	99.4	
Leishmania species	*L. infantum*	36	12.0 (8.0-17.0)	95.4	0.09	23	8.0 (4.0-14.0)	95.9	P<0.001
	*Leishmania* spp.	25	8.0 (4.0- 14.0)	96.7		9	15.0 (.5.0-48.0)	99.4	
	*L. infantum*, *L. tropica*	2	10.0 (8.0-12.0)	97.1		2	1.0 (0.0-1.0)	-	

**NR**=not reported, **NE**= not enough studies

**Figure 2.**
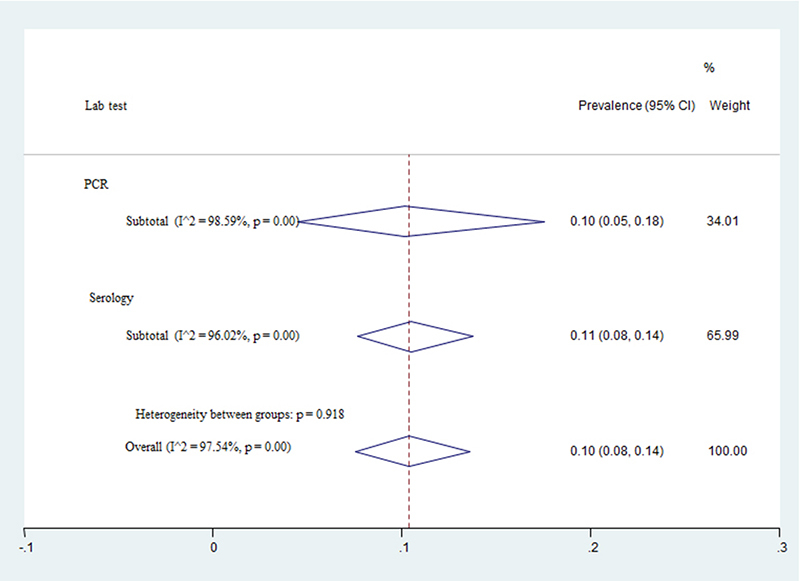
Forest plot for the prevalence of Leishmania infection in cats by PCR and serology tests.

The pooled prevalence of FLI based on a random effect meta-analysis ( 𝐼 2 =97.54 , 𝑃<0.001) was estimated at 10% (95% CI: 8%-14%). The estimate of prevalence based on seropositivity (11%), was significantly higher than PCR positivity (10%) (z=0.01, p=0.92) (Figure 2).

**Figure 3.**
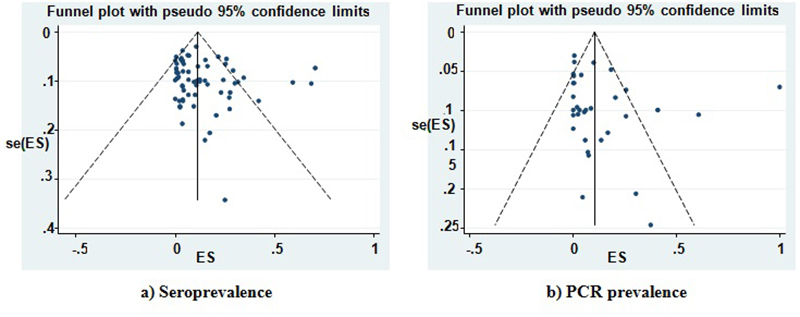
Funnel plot for seroprevalence (**a**) and PCR prevalence (**b**) of Leishmania infection in cats.

 Not only funnel plot but also Egger's test found no evidence a heterogeneity among effect size of studies for seroprevalence (b=1.36, p= 0.180 ) and PCR prevalence (b= 0.16 , p= 0.875) (see Figure 3).

## Discussion

Zoonotic VL (ZVL) is a zoonosis that occurs in the Old and New World. Research studies on FLI are limited but have become more numerous internationally in recent years, especially in Brazil [23]. In this study the highest numbers of reported studies (30.8%) were found in Brazil, where leishmaniasis is a major public health problem. Brazil is one of the countries with the highest prevalence and widest geographical distribution of the disease [2, 24]. 

In the current study, the overall prevalence of FLI was estimated to be 10%. The relatively high prevalence of infection in cats demonstrates a similarity with dogs exposed to the leishmanial infections in some endemic areas [25-28 ]. A larger prevalence of L. infantum infection in dogs compared to cats is related to the immune-system differences in these two species and a more efficient Th1 immune response in cats compared to dogs [25,28]. The different FLI reports and the increased cat populations in diverse areas highlight the ability of these animals to maintain and spread the infection in natural and urban environments [28]. Overall, little information is available on the adaptive immune response of cats naturally exposed to L. infantum infection and mechanisms responsible for susceptibility or resistance of feline hosts. However, some evidence suggests that large numbers of clinical cases of FLI are reported in cats that are probably immunocompromised [8], although it seems that asymptomatic cases have an immunocompetent condition and act as cryptic reservoir hosts.

Based on our findings, FLI in the mixed-type /breed cats were higher than in other feline types/-breeds. The role of domestic cats has been controversial in leishmaniasis epidemiology because they live in close contact with humans. Domestic cats can act as primary, secondary or accidental hosts [7, 28]. 

Seroprevalence rates from 0.9% to 28.5% and PCR detection rates between 0.43% and 30% have been reported in some regions and countries such as Spain, Portugal , France, and Italy in Southern Europe, as well as in North Africa, Iraq, Iran, Turkey and Central and South America, where canine leishmaniasis is endemic [29-36]. 

In our study, both seroprevalence (24%) and PCR prevalence (21%) of FLI were found higher in Italy than in other countries. Moreover, our data show the high seropraevalence rate (12.2%) of FLI in Southern European countries including Italy, Spain, Portugal, and Greece. However, this could be justified by the increased finding of active cases in cats, development of simple and rapid diagnostic tests and elevated rate of disease prevalence in these countries. 

Diagnosis is usually based on the results of cytology, histopathology, immunohistochemistry (IHC), culture, serology and PCR. Apart from the advantages and limitations inherent to each of these methods, their diagnostic value depends on many factors, including the biological sample being used, the reagents and the particular technique employed. 

In our study the most common diagnostic laboratory method was IFAT (38.5%). IFAT and ELISA are the most common serological techniques used for diagnosis and for clinical and research studies on canine and feline leishmanial infections [10-16]. In areas endemic for Trypanosoma spp. or other Leishmania spp., cross reactions with L. infantum must be taken into account for interpretation of serological tests [16]. However, some attention is needed before confirming leishmaniasis with IFAT in cats, whereas cats that present clinical symptoms for leishmaniasis but are found negative by IFAT should be subjected to other serological tests or complementary diagnostic tools such as WB and PCR [7]. WB analysis, a qualitative serological method, distinguishes the molecular weight of the L. infantum antigens stimulating antibody production, but is less frequently used for the diagnosis of leishmaniasis [37]. One potential application of the WB method is the discrimination between subclinical and clinical infections [38].

Three different species of Leishmania have been found in cats in Brazil: L. amazonensis [39], L. braziliensis [40] and L. infantum [34, 41]. Five Leishmania species have been reported in cats worldwide, although most cases involved L. infantum [8]. This is in agreement with our findings in the current study in which approximately 63% of species were L. infantum.

In conclusion, our data provide substantial evidence that cats can be considered sentinel reservoir hosts at least in endemic foci of zoonotic visceral leishmaniasis. Moreover, the current data demonstrate enzootic stability of FLI in several countries of the world particularly in some European countries. Furthermore, our results show the most common lab method for diagnosing FVL is the IFA test. In general, control of cat populations is recommended to reduce the transmission of Leishmania spp. among human populations in the endemic areas especially among nomadic tribes [42].

### Abbreviations

Not applicable.
